# A Review on Dry Eye Disease Treatment: Recent Progress, Diagnostics, and Future Perspectives

**DOI:** 10.3390/pharmaceutics15030990

**Published:** 2023-03-19

**Authors:** Himangsu Mondal, Ho-Joong Kim, Nijaya Mohanto, Jun-Pil Jee

**Affiliations:** 1 Drug Delivery Research Lab, College of Pharmacy, Chosun University, Gwangju 61452, Republic of Korea; 2 Department of Chemistry, Chosun University, Gwangju 61452, Republic of Korea

**Keywords:** dry eye, ocular, drug delivery, contact lens, cyclosporin A, biomarkers, biosensors

## Abstract

Dry eye disease is a multifactorial disorder of the eye and tear film with potential damage to the ocular surface. Various treatment approaches for this disorder aim to alleviate disease symptoms and restore the normal ophthalmic environment. The most widely used dosage form is eye drops of different drugs with 5% bioavailability. The use of contact lenses to deliver drugs increases bioavailability by up to 50%. Cyclosporin A is a hydrophobic drug loaded onto contact lenses to treat dry eye disease with significant improvement. The tear is a source of vital biomarkers for various systemic and ocular disorders. Several biomarkers related to dry eye disease have been identified. Contact lens sensing technology has become sufficiently advanced to detect specific biomarkers and predict disease conditions accurately. This review focuses on dry eye disease treatment with cyclosporin A-loaded contact lenses, contact lens biosensors for ocular biomarkers of dry eye disease, and the possibility of integrating sensors in therapeutic contact lenses.

## 1. Introduction

Dry eye disease (DED) is an illness of the preocular tear film that occurs due to injury to the eye surface and is associated with signs of ocular discomfort. It is also known as keratoconjunctivitis sicca, sicca syndrome, keratitis sicca, dry eye syndrome (DES), xerophthalmia, dysfunctional tear syndrome, ocular surface disease, or simply dry eyes. “Sjögren’s syndrome” is a form of DES where the eyes do not produce enough tears [1].

The International Dry Eye Workshop (2007) defined dry eye as a multifactorial disease of the tear film and ocular surface that results in symptoms of discomfort, visual disturbance, and tear film instability, with potential damage to the ocular surface. It is associated with ocular surface inflammation and increased tear film osmolarity [2]. A normal human tear film comprises water, lipids, mucins, electrolytes, proteins, and vitamins. Along with goblet cells, the meibomian and lacrimal glands produce tears that maintain a normal ocular environment by removing debris, lubricating the eye, and protecting the eye from infection [3]. Recently, it was found that dry eye is an inflammatory disease that shares many features with autoimmune diseases. The pathogenesis of dry eye may be due to stress on the ocular surface (infection, environmental factors, endogenous stress, genetic factors, and antigens) [4,5]. DES is a chronic disease, particularly among older people, but proper treatment decreases symptoms and, eventually, ocular damage [3].

The prevalence rate is 5–50%, which may be up to 75% in adults over 40 years old, with women being the most affected [6]. For younger adults aged 18–45 years, only 2.7% may develop DED [7]. It has an economic impact, ranging from $687 to $1267 annually, depending on the severity of the disease. DED costs the US economy approximately $3.8 billion [7,8]. These costs include prescription drugs, over-the-counter products, and punctual plug placement [9].

**Figure 1:**
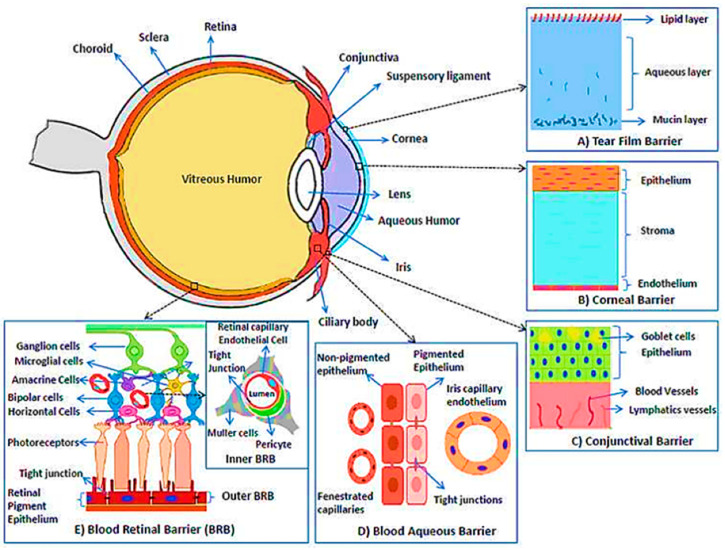
Structure of the eye and different barriers for drug delivery. (**A**) Tear film barrier. (**B**) Corneal barrier. (**C**) Conjunctival barrier. (**D**) Blood-aqueous barrier. (**E**) Blood-retinal barrier (BRB). Reprinted with permission from reference [12]. Copyright 2017 Elsevier.

The eye is a complex organ with various anatomical and physiological barriers (Figure 1). These complex structures make ocular drug delivery challenging for scientists [10]. Available ophthalmic dosage forms include eye drops, ointments, and suspensions. Among these preparations, eye drops hold the major part (>90%) [11]. A healthy human has a 7–30 μL tear with a 0.5~2.2 μL/min turnover rate. The restoration time of the tear film is 2–3 min [12]. Thus, approximately 1–5% of the applied drug reaches the intended tissue. The ineffectiveness of this treatment necessitates repeated application at higher concentrations, which affects the patient’s normal routine [13]. Numerous innovative drug delivery techniques have been developed to enhance drug residence duration and cross the cornea, but all have limitations [14,15]. Therefore, a unique drug delivery method can enhance patient compliance while increasing drug bioavailability and reducing systemic exposure to improve clinical outcomes [13]. Drug delivery using contact lenses is an intriguing field of research because of its exclusive features, such as prolonged wear, simple end of therapy (by removing the contact lens), and greater bioavailability (>50%) than eye drops, as established by numerous studies [16,17]. Patients’ high compliance may be achieved with drug-loaded contact lenses by excluding multiple drug dosing, particularly for contact lenses worn mainly for vision improvement [18,19].

As the solitary sensory organ of the human optical system, the human eye contains a wealth of important chemical, physical, and biological biomarkers related to human health. Consequently, it has become an important research topic, propelling the rapid development of soft electronic systems for eye research [20]. Biosensor-integrated contact lenses can be a good choice for pliable and wearable therapeutic devices [21]. They have sensing gears to track eye conditions, such as intraocular pressure (IOP) and tear fluid constitution [22]. The biosensing functionalities of contact lenses have become possible with advancements in device downsizing for microcircuits, microsensors, and other microscale devices [20]. There are two major groups of sensors for sensing tear fluid: chemical (biomolecules, metabolites, and electrolytes) and physiological (wrinkling behavior, tear production, IOP, and temperature) sensors [23,24]. Electrochemical sensing has a higher sensitivity and temporal resolution than fluorescence-based sensing using colorimetric assays [25,26]. In the case of DED, the tear production rate can be determined colorimetrically using microfluidic cells with a coloring dye if implanted in contact lenses [27].

This review aimed to (1) evaluate the current treatments for DED, (2) assess the potential of contact lens-based drug delivery as a new treatment for DED, covering the features of the current technologies and their pros and cons, (3) describe various biosensor technologies that can identify pathological eye characteristics, and (4) provide a future perspective of biosensor-fused contact lens-based drug delivery.

## 2. Treatments of Dry Eye Disease

As DED is a chronic disease, the treatment may take a long time to get a positive result, which can be achieved in different ways (Table 1). The treatment involves a hierarchy approach according to disease severity and information related to (subclinical) inflammation of the ocular surface, meibomian gland dysfunction, and/or associated systemic disease [28]. Cigarette smoking, air conditioning, and dry heating air increase the risk of DED, which must be avoided [5]. Currently, many drugs for DED treatments are in clinical trials (Table 2).

### 2.1. Weakness of Existing Treatments

The available dosage forms for DED are primarily eye drops or emulsions. After application, they tend to rapidly enter the nasolacrimal duct with a low turnover rate and short restoration time. The drug is then eliminated through lymphatic flow and conjunctival blood. As a result, only 1–5% of the drug administered is available for absorption by the target tissue. The bioavailability of lipophilic and hydrophilic drugs in eye drops is less than 5% and 0.5%, respectively. Frequent dosing with highly concentrated drugs [60] is required to overcome this limitation. This may be responsible for poor patient compliance, especially for chronic ocular disease, such as DES [10,61]. The fraction of the delivered drug that enters the systemic circulation escapes first-pass metabolism and enters all major organs, with potential side effects [60].

For 18.2–80% of patients, there is a chance of microbial contamination when administering eye drops due to contact between their face, eyes, or hands and the dispensing tip of the eye drop container. The delivery of the same number of drops is not always possible, and approximately 11.3–60.6% of patients fail to do this. Moreover, the amount of drug expelled from the eye drop bottle is not constant and depends on the force applied on the bottle surface, which may lead to dose variations [10,62]. Therefore, it is not possible to deliver the correct amount of drug irrespective of careful handling of conventional eye drops, and that might fail to gain patients’ satisfaction and may lead to poor clinical outcomes [10,63].

### 2.2. Contact Lenses as an Alternative DED Treatment

**Figure 2:**
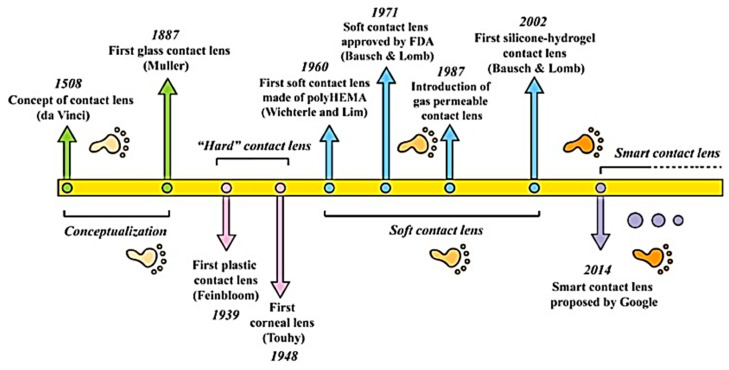
The milestones of contact lens development. Reprinted with permission from reference [24]. Copyright 2021 John Wiley and Sons.

An innovative dosage form is required to overcome the existing limitations of DED treatment. In this regard, contact lenses are a suitable candidate. Contact lenses have undergone numerous advancements and modifications (Figure 2).

Contact lenses, which are thin, curved, plastic lenses, are worn to protect the eye or correct vision. In 1965, Sedlacek first introduced contact lenses as vehicles for ocular drug delivery. Hydrogels and silicone hydrogels are suitable materials for drug-laden contact lenses. 2-hydroxyethyl methacrylate (HEMA) is polymerized to obtain hydrogel contact lenses with low oxygen permeability [64]. The introduction of various hydrophilic monomers amplifies the water content and subsequent oxygen permeation. HEMA-based contact lenses can be worn for less than 6 days [65]. However, silicone hydrogel lenses can be worn for 29 days and have high oxygen permeability [66,67]. Compared with eye drops, contact lenses extend the residence time of drugs in the eye from 2 min to 30 min, resulting in improved drug bioavailability in the cornea [68]. Potential side effects are also reduced as drug exposure in the systemic circulation is minimized [69].

Therapeutic or drug-eluting contact lenses can be excellent substitutes for treating eye diseases. With increased residence time of the drug in front of the cornea, the bioavailability of the drug also increases up to approximately 50%, which in turn can increase drug efficacy and abate systemic side effects [70,71]. This platform may increase the patient’s compliance with single drug administrations, especially for vision correction patients who wear contact lenses [10,18]. Millions of people suffer from different types of ocular diseases. There are three purposes for developing a therapeutic contact lens to provide the loaded drug for slow and longer release periods. First, a “comfortable lens” ensures longer wearing of contact lenses for dry eyes. Second, “patients’ compliance” by providing an easier way to maintain treatment effectiveness. Third, “bandage lenses” for managing postoperative complications, corneal wound healing, and viral corneal erosion. Anti-inflammatory or antimicrobial drugs may be incorporated into the lens, which releases the drug throughout the treatment period (1–30 days) [11]. Contact lens-based delivery of drugs for the treatment of DED are listed in Table 3.

**Figure 3:**
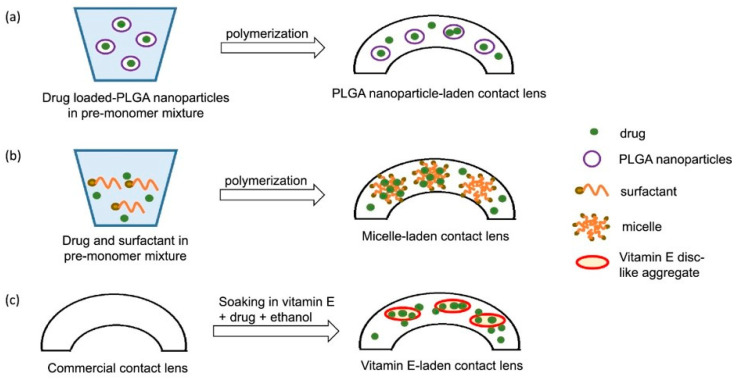
Hydrophobic drug controlled release techniques from soft contact lenses. (**a**) Nanoparticles loaded contact lenses. (**b**) Contact lenses with micelles. (**c**) Vitamin E fused contact lenses. Reprinted with permission from reference [72]. Copyright 2020 Elsevier.

The first technique for drug loading to the contact lens was soaking the contact lens in the drug solution, but within 1–3 h, almost all the loaded drug was released. Several procedures have been introduced to design drug-loaded contact lenses (Figure 3), including the diffusion of vitamin E barriers, molecular imprinting, prolonged drug release by ionic interactions, drug-loaded implants, colloidal micro-and nanoparticles with drugs, and supercritical fluid technology, to avoid this limitation [17].

**Table 3:** DED treatment with contact lens drug delivery systems.

Drug Molecules	Contact Lens Type	Drug Loading Method	Duration	References
Cyclosporine A	Hydrogel and silicone hydrogel	Soaking	1 day (hydrogel) 15 days (silicone hydrogel). Pre-soaking with vit. E increases time release to 30 days	[73]
Hyaluronic acid	Hydrogel and silicone hydrogel	Soaking	1 h	[74]
Phospholipids	Silicone hydrogel	Soaking	10 h	[75]
Dexamethasone	Silicone hydrogel	Soaking	2 weeks–3 months	[76]
Dexamethasone	Silicone hydrogel	Soaking	7 days	[77,78]
Ap_4_A (Secretagoge)	Hydrogel and silicone hydrogel	Soaking	5–6 h	[41,79]
Betaine (Osmoprotectant)	Silicone hydrogel	Soaking	10 h	[41,80]
Polyvinilpyrrolidone	Hydrogel	Polymerization	30 days	[81]
Hyaluronic acid	Hydrogel and silicone hydrogel	Polymerization	21 days (hydrogel), 49 days (silicone hydrogel)	[82]
Polyvinilpyrrolidone	Hydrogel	Polymerization	30 days	[81]
Diclofenac	Hydrogel	Copolymerization	7 days	[83]
Dexamethasone	Hydrogel	Copolymerization	50 h	[84]
Diclofenac	Hydrogel	Copolymerization	14 days	[85]
Cyclosporine A	Hydrogel	Nanoparticles (Brij surfactants)	20–30 days	[86,87,88]
Dexamethasone	Hydrogel	Nanoparticles/soaking	50 h	[89]
Hydroxypropyl methylcellulose	Silicone hydrogel	Molecular imprinting	60 days	[90]
Hyaluronic acid	Hydrogel and silicone hydrogel	Molecular imprinting	24 h	[91]
Diclofenac	Hydrogel	Molecular imprinting	6 days	[79]

### 2.3. Contact Lens-Based Drug Delivery for DED

Dry eye treatment aims to relieve symptoms, restore the ocular surface and tear film, enhance visual perception and quality of life, and correct causal defects. As dry eye disease is multifactorial, several therapies have been suggested for its management, including drug delivery through contact lenses. Cyclosporin A (CsA) can be loaded into the contact lens for DED treatment.

#### 2.3.1. Advantages of CsA

The underlying pathogenesis of DED lies in the infiltration of T-cells in the conjunctiva tissue, as well as the presence of proteases and cytokines in the tear fluid, which was the primary reason for presenting the use of immunomodulatory agents, such as corticosteroids, doxycycline, and CsA, and to treat DED. The FDA has authorized CsA emulsion for the treatment of dry eye, and clinical trials have confirmed CsA’s effectiveness and safety. CsA appears to be a viable therapy for DED [92].

*Tolypocladium inflatum* is a CsA-producing fungus [93]. The natural product CsA is an immunosuppressant drug, and its immunosuppressive activity was first observed in 1976 [94,95]. It works by inhibiting the calcineurin–phosphatase pathway via complex formation with cyclophilin and thus reduces the transcription of T cell-activating cytokines, such as interleukin-2 (IL-2) and IL-4. It was the first drug approved by the US FDA for DED by topical application [37]. Additionally, CsA reduces the expression of proinflammatory chemokines and cytokines, such as IL-1β, IL-6, tumor necrosis factor-alpha (TNF-α), vascular cell adhesion molecule 1, and intercellular adhesion molecule 1, in benzalkonium chloride-mediated DED [96,97]. It also shelters the conjunctival epithelial cells of humans through anti-apoptotic activity, increases the density of conjunctival goblet cells, and improves the integrity of the corneal surface through immunomodulatory actions [98,99]. Goblet and epithelial cell apoptosis are related to a decrease in interferon-γ expression, resulting from CsA reduction in T-cell involvement and activation [100,101].

CsA for DED treatment is advantageous over other corticosteroids in several ways. First, the effect is reversible after therapy. Second, it has a very low systemic absorption rate. Third, no critical side effects were observed. These pharmacokinetic parameters are crucial because long-term treatment is essential for chronic illnesses, such as dry eye. Moreover, the advantages of CsA begin after 30 days of treatment, and a 90 days course of treatment appears to be required [92].

#### 2.3.2. In Vitro CsA Release from Contact Lenses

CsA is a cyclic peptide of non-ribosomal origin consisting of 11 amino acids with a single d-amino acid. The cyclic structure consists of hydrogen bonds. The low aqueous solubility of CsA is due to this property and, thus, different cellular absorptions [102]. Several studies have demonstrated different techniques and construction materials for incorporating CsA into contact lenses (Table 4). CsA was loaded into silicone hydrogel contact lenses and hydrophilic poly-HEMA (p-HEMA) lenses by simple soaking. As CsA is a highly lipophilic drug, it has a higher affinity for lipophilic silicone-rich phases than the hydrophilic p-HEMA phase. Therefore, the partition coefficient is higher in silicone hydrogel contact lenses than in hydrophilic p-HEMA lenses. The in vitro release durations of CsA from silicone hydrogel contact lenses and hydrophilic p-HEMA contact lenses were approximately 15 days and 1 day, respectively [41]. In addition, the vitamin E barrier is used to slow and extend drug release (Figure 3c) with minimal impact on vital lens properties, such as light refraction and oxygen permeability [17,77]. The in vitro release of CsA was prolonged for approximately 1 month with a 20% loading of vitamin E into the silicone hydrogel contact lenses.

**Figure 4:**
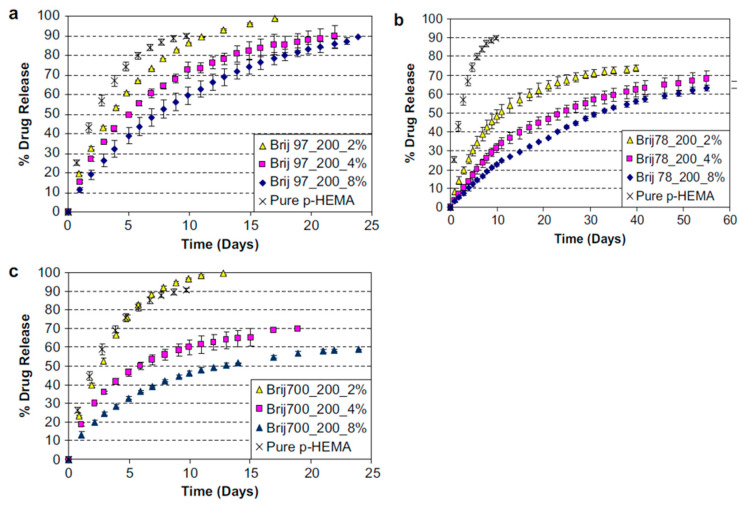
Influence of surfactant on cumulative release of CsA (50 mg/contact lens). The contact lenses consisted with different surfactants: (**a**) Brij 97, (**b**) Brij 78, and (**c**) Brij 700. Adapted from reference [86]. Copyright 2008 Elsevier.

**Figure 5:**
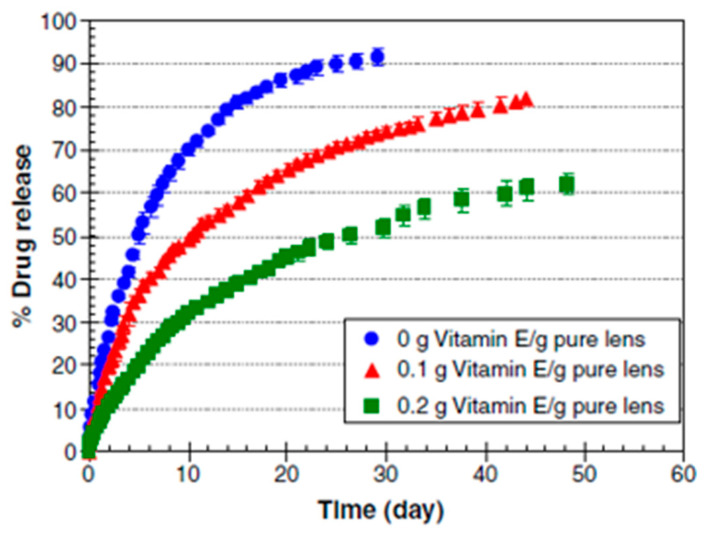
Effect of vitamin E on cumulative release of CsA from ACUVUE^®^ OASYS™ contact lenses. Adapted from reference [73]. Copyright 2011 Elsevier.

The use of surfactant in the p-HEMA contact lens polymerizing mixture forms micellar aggregates by creating hydrophobic sites inside the gel, where the hydrophobic CsA may preferentially partition. Kapoor et al. (2009) developed Brij surfactant-laden p-HEMA hydrogel contact lenses using CsA. Brij 97 and Brij 98 surfactants showed slow and longer CsA release of approximately 20 days. Brij 78 surfactant seemed most capable of delaying the release of CsA from p-HEMA contact lenses for more than 30 days (Figure 4) [41,86]. Peng and Chauhan (2011) showed that the incorporation of vitamin E extended the release of CsA for more than 30 days (Figure 5) [73]. Desai et al. (2021) developed a contact lens with graphene oxide to incorporate the hydrophobic drug CsA by the soaking method and evaluated its in vitro drug release. They demonstrated that the increased drug uptake did not change the optical or swelling characteristics [104].

#### 2.3.3. In Vivo Biological Activity of CsA Contact Lenses

**Figure 6:**
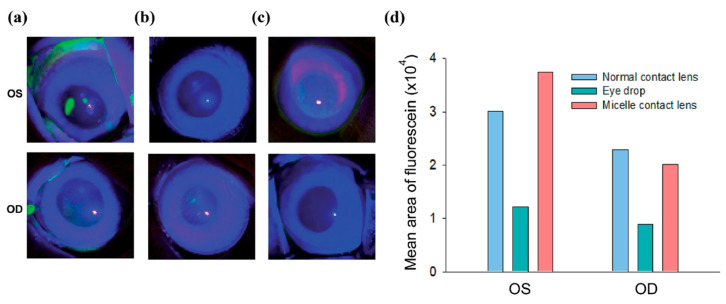
Corneal inflammation inspection by corneal fluorescein staining followed by (**a**) normal contact lens wear in OD, (**b**) CsA eye drop administration, and (**c**) CsA/C-HA micelle contact lens wear in OD. (**d**) The ROI (region of interest—ROI) values for fluorescein staining (oculus dexter—OD, oculus sinister—OS). In case of micelle CL, the OS did not contain any CsA, so the fluorescein staining showed increased intensity than the OD. Oculus dexter is abbreviated as OD, meaning right eye, and oculus sinister is abbreviated as OS, meaning left eye. Reprinted with permission from reference [103]. Copyright the Royal Society of Chemistry.

**Figure 7:**
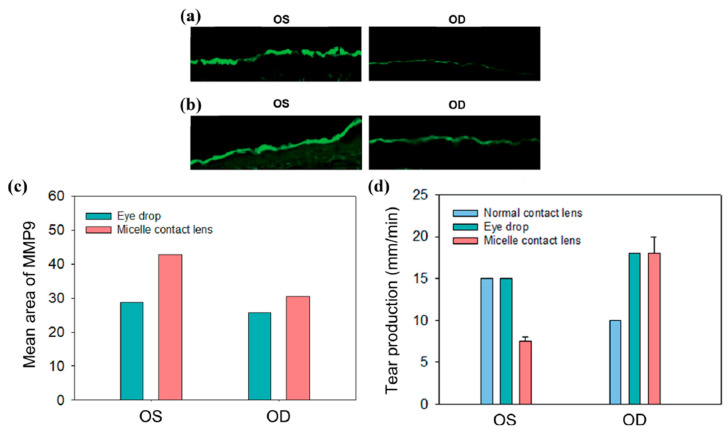
Analysis of corneal inflammation using the MMP9 DED marker (corneal immunofluorescein staining, green color) after (**a**) CsA eye drop application, and (**b**) CsA/C-HA micelle contact lens wear. (**c**) ROI values for MMP9. (**d**) Tear production by the contact lens-wearing (OD) and control (OS) eyes at day 7. Tear production was increased in the treated OD groups. Oculus dexter is abbreviated as OD, meaning right eye, and oculus sinister is abbreviated as OS, meaning left eye. Reprinted with permission from reference [103]. Copyright the Royal Society of Chemistry.

Mun et al. (2019) showed that CsA release from the contact lens in the rabbit eye produced a significant DED improvement (Figure 6). The DED was induced in rabbits with 3-concanavalin A injections (Con A, sigma L7647). The contact lens was able to release CsA for up to 7 days. Corneal immunofluorescence staining for MMP9 (a DED marker) was performed to confirm the treatment outcomes (Figure 7) [103]. A decrease in the MMP9 intensity was observed for the right eyes (OD) treated with eye drops and CsA/C-HA micelle CL compared to control group (OS). Desai et al. (2022) observed that rabbits recovered quickly from DED with a CsA graphene contact lens and a higher amount of CsA in the corneal fluid for a long time [104].

## 3. Challenges of Contact Lenses in Drug Delivery

Scientists have succeeded in prolonging drug release using contact lenses; however, critical lens properties, such as oxygen permeability, swelling, ion permeability, optical transparency, tensile strength, issues during monomer extraction, high burst release, sterilization, and storage, are yet to be addressed [105]. Corneal damage and infections related to the extended wearing of contact lenses for chronic diseases, such as DED, must be considered [13,91,106]. Contact lenses integrated with drug delivery systems are classified as combination medical products, which may delay the approval of new materials. The polymerization process and the chemical and physical properties of the generated hydrogel may be influenced by drug properties (the physical and chemical). Bacterial resistance and ocular toxicity may arise from long-term drug release from contact lenses [107].

A major limitation of contact-lens drug delivery is the initial burst release of the drug, which can lead to potential systemic toxicities. Researchers have employed various technologies, such as p-HEMA hydrogel, implants, molecular imprinting, use of vitamin E, and incorporation of drug-loaded nanoparticles, to minimize it [108]. Recently, bioelectronics and biosensors have been intensely utilized to monitor health conditions in real-time for chronic diseases and coronavirus disease 2019 (COVID-19). These multifunctional sensors process physiological signals to digital data without disturbing normal biological activities and can dramatically enhance therapeutic outcomes. These small devices can help deliver medicines more precisely, and personalization is possible [109]. Biosensors can be integrated with contact lenses to monitor disease conditions continuously via specific biomarkers for ocular diseases, improving lens properties and minimizing drug related systemic toxicities.

## 4. Biosensors Integrated Contact Lens

**Figure 8:**
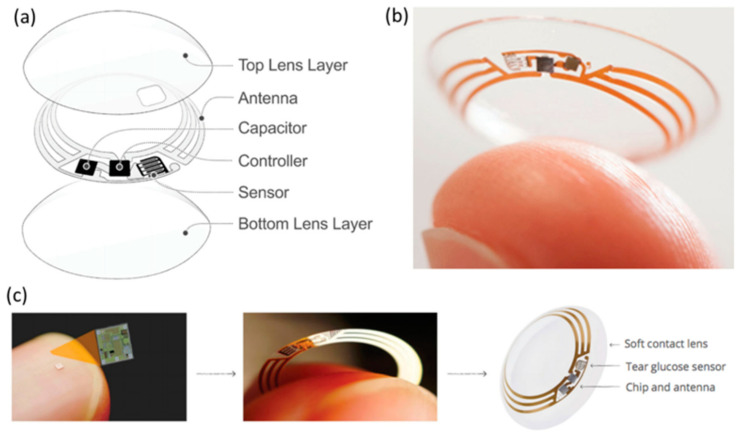
The contact lens sensor for tear glucose measurement, developed by Google and Novartis. (**a**) A diagram of the contact lens sensors. (**b**) The model contact lens sensor. (**c**) The wireless chip for the sensor. Reprinted with permission from reference [114]. Copyright 2021 John Wiley and Sons.

A biosensor is a diagnostic device used to sense a chemical substance that combines a biological element with a physicochemical indicator (Figure 8) [110,111,112,113]. The delicate biological components, such as enzymes, tissue, antibodies, organelles, cell receptors, and nucleic acids, are biologically obtained elements that bind with, interact with, or distinguish the analyte under experiment. The detector transforms one signal into another. It works through different mechanisms, for example, piezoelectric, electrochemiluminescence optical, and electrochemical, through the reaction of the analyte and biological sample to measure and quantify. A reader is connected to the display to show the results simply [99].

The human eye carries important chemical, physical, and biological data related to human health. Hence, it appears to be a vital research target that drives the rapid growth of soft electronic systems for diagnosing various diseases of the eye and other organs. As wearable and flexible medical devices, contact lenses have a significant capacity to support the analysis and treatment of ocular diseases [20].

### 4.1. Tear Film Biomarkers for DED

The concentration of the tear fluid constituents was related to its concentration in the blood (Table 5). DED is a multifactorial inflammatory disease characterized by tear film instability, ocular discomfort, visual disturbances, inflammation, and increased tear osmolarity. Research on tear film biomarkers has been increasing to identify diagnostic tools for DED or monitor treatment outcomes in clinical trials. In the last 5 years, numerous studies have been conducted on tear fluid biomarkers (Table 6) in DED [115]. Physicians face difficulty selecting suitable treatment options because of the lack of sufficient tools for observing and monitoring patient responses. Recent studies have investigated chemokines, cytokines, growth factors, neuromodulators, mucins, and lipids to find protein profiles that can be suitable biomarkers for DED [116]. Aluru and colleagues, found that lysozyme proline-rich protein 4 is downregulated in several types of DED, suggesting that this protein is a potential biomarker for DES [117]. Zhou and colleagues reported the upregulation of ∝-1 acid glycoprotein 1, ∝-enolase, calgranulin B, calgranulin A, and calgizzarin. Furthermore, four downregulated proteins, including lipocalin-1, prolactin-inducible protein, lysozyme, and lactoferrin, were also found in DED patients. Recently, different research groups have anticipated other proteins related to DED, regardless of the probable biomarkers under investigation [118]. For instance, malate dehydrogenase 2 activity increased, but mucin (MUC)5AC activity decreased. On the other hand, neuromediators, such as neuropeptide Y (NPY), calcitonin gene-related peptide (CGRP), nerve growth factor (NGF), and vasoactive intestinal peptide, have been identified as possible biomarkers for DED due to their association in clinical studies [119]. In DED, NGF levels were increased, while NPY and CGRP were found to decrease in tear fluid. Chhadva et al. (2015), reported higher tear serotonin concentrations in patients with DED [120].

The levels of chemokines and cytokines in tears play an important role in DED. Many studies have been conducted to identify a complete tear profile. Some inflammatory chemokine/cytokine levels (such as TNF-α, IL-1, IL-1RA, IL-6, metalloproteinase (MMP)-9, IL-8/C-X-C motif ligand (CXCL) 8, IL-17A, IL-22, interferon-γ, IP-10/CXCL10, MIG/CXCL9, I-TAC/CXCL11, macrophage inflammatory protein (MIP)-1β/CCL4, MIP-1α/CCL3, and RANTES/CCL5) are remarkably elevated in tears of patients with DED [115], while endothelial growth factor is decreased [128,129,130] with an increase in disease severity. Measurement of MMP-9 in tears has been proposed as a delicate technique for DED severity determination [131,132]. Some researchers have found that MMP-9 increases in the tear fluid of patients with DED [124,133,134,135]. Several studies have been conducted to identify the properties of tear lipids secreted by the meibomian gland in patients with DED. Compositional differences in the DED patient reflex tear metabolomic profile were revealed for N-acetylglucosamine, cholesterol, creatine, glutamate, amino-n-butyrate, acetylcholine, choline, arginine, glucose, phosphoethanolamine, and phenylalanine levels [126]. Willshire C et al. found that the basal tear osmolarity increases in DED compared to that in the control group [127]. Therefore, it may be a useful marker for DED. Moreover, correlated biomarkers in tears, such as cytokine profiles, have been anticipated for the initial diagnosis of the COVID-19 [136,137].

### 4.2. Contact Lens Sensors for Sensing of Tear Fluid Biomarkers in DED

**Figure 9:**
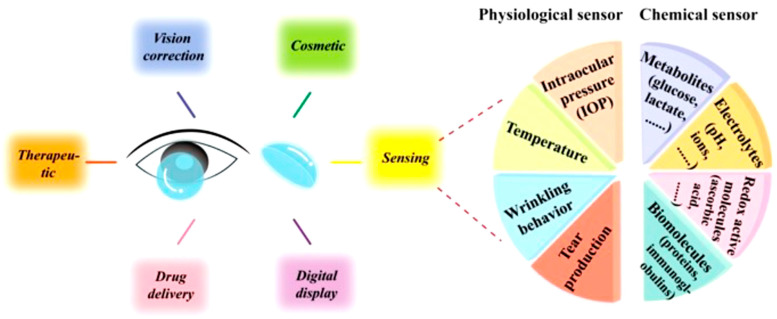
Sensing capability of contact lens sensors. Reprinted with permission from reference [24]. Copyright 2021 John Wiley and Sons.

Tear osmolarity is the only clinically established parameter directly associated with dry eye severity [103]. Human tears’ chemical components (biomarkers) include proteins, electrolytes, lipids, urea, L-lactic acid, cholesterol, ascorbic acid, and many metabolites. If their concentrations in tears are known, then their concentrations in the blood could be correlated. Therefore, concurrently analyzing their concentrations in tears provides important physiological data that can improve treatment outcomes and anticipation of some illnesses [20]. Identifying appropriate biomarkers for specific diseases is a major challenge and an ongoing process. Once a biomarker is identified, it is tested for biosensor applications that vary from functionally integrated (contact lens) to on-chip sensors (Figure 9) [116]. Currently, it is possible to measure multiple parameters, such as the glucose level and IOP, using a single contact lens that integrates many sensors [138]. The number of illnesses that can be tracked and identified with contact lens biosensors will increase as sensing technology advances. Contact lenses can naturally gather tear components during wear and may be examined thereafter. It would be feasible to detect the existence and progression of certain diseases by combining the detection of certain biomarkers, such as cancer or dry eye [85]. Some electrochemical sensors have already been developed to identify several biomarkers (Table 7) in tear fluid to monitor the condition of patients with DED. Thus, integrating these sensors into a therapeutic contact lens can continuously track DED progress.

## 5. The Future Perspective of Biosensor Fused Contact Lens

The concept of biosensors in contact lenses is recent and unique. The implementation of biosensors in a contact lens can measure specific parameters even during sleep, enabling the analysis of the pattern of disease conditions at night or during sleeping hours [138]. Most contact lenses can only detect one biomarker in the eye, such as glucose, lactic acid, K^+^, or Ca^2+^. The detection of multiple chemical components in real-time increases the biomedical utility of contact lenses [139]. Most existing sensory systems lack the ability to power themselves. As natural sunlight is readily available for energy conversion, flexible photovoltaic self-powered technology can replace standard power supply modes in contact lenses. Photovoltaics will be a future trend in inflexible and stretchable electronics because of these and other characteristics [140]. More chips and interconnects must be added to the device to increase the performance and multifunctionality of contact lenses. The use of transparent materials, such as graphene, carbon nanowires, and indium tin oxide, will make this work easier. The shrinking of chips incorporated in the system for data storage, data transfer, and circuit powering has become increasingly significant, driving researchers and industrial suppliers to produce next-generation chips with multiplexed capabilities. Furthermore, when the entire circuit was scaled down, the sensitivity of the device was considerably diminished, particularly with respect to the size of the sensing electrodes. One possible solution to these issues is to use active sensors, such as field-effect transistors and complementary metal-oxide-semiconductor sensors, which have remarkable sensitivity and are sub-micrometers in size [20,141]. The concentration of tear biomarkers is low, which necessitates the use of highly sensitive biosensors. In addition, the development of sensitive biosensors is very expensive. Tear makeup varies significantly across and between individuals. The lag time caused by biomarker diffusion from the tear fluid to the implanted biosensor can affect the treatment outcomes. When the biosensor is attached to a contact lens, it becomes thicker and can cause patient discomfort [142].

Contact lenses have shown enormous potential in biomedicine owing to their features, such as real-time and non-invasive diagnostics and drug delivery. Multifunctional and integrated contact lenses can record physiological data regarding eye problems more efficiently than earlier approaches, reducing the need for human illness treatment. It offers great promise as an everyday medical device for the reliable measurement of ocular response to ophthalmic drugs and surgical procedure evaluation. Contact lenses represent technical and material advances that will pave the way for the next generation of precision medicine-based products [100]. Soon, it will be possible to combine biosensors in a medicated contact lens that will release the drug and monitor the overall disease condition simultaneously. No study has reported the electrical control of drug release from contact lenses using simultaneous biometric analysis [143]. Future biosensors may control drug release from the contact lens in response to a patient’s need.

## 6. Conclusions

Drug-loaded contact lenses are a promising option for treating chronic ocular diseases. In the last few years, a number of innovative contact lens drug-delivery systems have been established that increase the drug-loading capacity and control the drug-release rate. Treatment of DED with a CsA-loaded contact lens has been successful in animal models. Further studies are required to confirm its feasibility in clinical trials. Contact lenses are not limited to drug-delivery devices, as they can also be used as a diagnostic tool. Contact lens sensor technology has gained popularity over the last decade, primarily owing to developments in the downsizing of electrical circuits and the discovery of several significant biomarkers in tear fluid. This sensor platform offers various advantages, including its non-invasive and constant biomarker-measuring properties. However, significant advancements in specificity, sensitivity, biocompatibility, integration with readout circuitry, and repeatability are still being made for such platforms to achieve feasibility. For example, a self-powered biosensor significantly simplifies the sensor layout. Furthermore, a better understanding of the relationship between illness and ocular biomarker concentration is necessary to develop practical multifunctional contact lens biosensors. This might pave the way for personalized therapeutic contact lenses with biosensors.

## Figures and Tables

**Table 1:** Dry eye disease treatments.

Medication	Description	Mechanism of Action	References
Artificial tears	Polyvinyl alcohol, povidone, hydroxypropyl guar, cellulose derivatives, and hyaluronic acid	Increase tear film stability. Reduce ocular surface stress. Improve contrast sensitivity and the optical quality of the surface.	[28,29,30,31,32,33]
Topical corticosteroids (loteprednol 0.5%)	Unpreserved corticosteroid eyedrops, instilled over a period of 2 to 4 weeks, improve the symptoms and clinical signs of moderate to severe dry eye disease.	Corticosteroids act by the induction of phospholipase A2 inhibitory proteins and inhibiting the release of arachidonic acid.	[34,35,36]
Cyclosporin A (CsA)	Topical application of CsA leads to increased production of tear fluid, possibly via local release of parasympathetic neuro transmitters. CsA eyedrops 0.05% (Restasis) were approved for the topical treatment of dry eye by the FDA in 2002.	CsA is an immunosuppressant that inhibits the calcineurin–phosphatase pathway by complex formation with cyclophilin, and thus reduces the transcription of T-cell-activating cytokines such as interleukin-2 (IL-2).	[37,38,39,40,41]
Tacrolimus/pimecrolimus	Appear to be as effective as CsA and are used in patients who cannot tolerate CsA	Inhibition of interleukin-2 gene transcription, nitric oxide synthase activation, cell degranulation, and apoptosis.	[5]
Tetracyclines	Bacteriostatic antibiotics with anti-inflammatory effect.	They reduce the synthesis and activity of matrix metalloproteinases, the production of interleukin-1 (IL-1) and tumor necrosis factor, collagenase activity, and B-cell activation.	[5,42,43]
Macrolides	Azithromycin 1% has been successfully used to improve meibomian gland function and symptoms, a reduction in bacterial colonization of the eyelid margins, and normalization of the meibomian gland secretion lipid profile.	Inhibition of bacterial protein biosynthesis by preventing peptidyltransferase from adding the growing peptide attached to tRNA to the next amino acid and also inhibiting bacterial ribosomal translation.	[44,45,46]
Omega fatty acids	Omega-3 and omega-6 are essential fatty acids for ocular surface homeostasis.	Omega-3 fatty acids work by blocking pro-inflammatory eicosanoids and reducing cytokines through anti-inflammatory activity.	[47]
Eyelid hygiene	Hot compresses, eyelid warming masks or goggles, infrared heaters, and eyelid massage improve eyelid margin morphology with a reduction in blocked meibomian gland excretory ducts, and an increase in tear film stability and lipid layer thickness of the tear film.		[48,49,50,51]
Punctal plugs	Temporary occlusion of the tear ducts by small collagen or silicone plugs (punctal plugs) is effective in patients with severe aqueous-deficient dry eye disease.		[36,52,53]
Lifitegrast (Xidra)	The U.S. Food and Drug Administration approved Xiidra (lifitegrast ophthalmic solution) for the treatment of signs and symptoms of dry eye disease, on Monday, 11 July 2016. Xiidra is the first medication in a new class of drugs, called lymphocyte function-associated antigen 1 (LFA-1) antagonist, approved by the FDA for dry eye disease. Xiidra is manufactured by Shire US Inc., of Lexington, Massachusetts.	Lifitegrast blocks the interaction of cell surface proteins LFA-1 and intercellular adhesion molecule-1 (ICAM-1), and is believed to inhibit T-cell-mediated inflammation in DED.	[54,55]
Vitamin A	Vitamin A is an essential nutrient present naturally in tear film of healthy eyes. Vitamin A plays an important role in production of the mucin layer, the most innermost lubricating layer of tear film that is crucial for a healthy tear film. Vitamin A deficiency leads to loss of mucin layer and goblet cell atrophy.	Vitamin A drops protect the eyes from free radicals, toxins, allergens, and inflammation.	[1,28,56]
Vitamin E	Vitamin E is a fat-soluble antioxidant that prevents the oxidation of fatty acids by reactive oxygen species. The retina is a lipid-rich environment and is bombarded by ultraviolet radiation. In cell culture, vitamin E has been found to enhance the antioxidant ability of lutein to protect retinal pigment epithelial cells from acrolein-induced oxidation.		[57,58]

**Table 2:** Clinical trials of DED drugs [59]. Copyright 2022 Elsevier.

Functions	Drug	Stage
A mucin-like glycoprotein	Lacritin	Phase II
	Lubricin	Phase II
Anti-inflammatory and/or immunosuppressive	Loteprednol etabonate 0.25% suspension	FDA-approved
	OCS-O_2_	Phase II
	A higher concentration of Cyclosporine	Phase III
	Tacrolimus (0.03%) eye drops	Phase IV
	Rapamycin (sirolimus)	Phase I
	EBI-005	Phase III
	Resolvin E1 analogues	Phase II
Biological components	Albumin 5%	Phase II
	Estradiol	Phase II
	*N*-acetylcysteine	Phase II
	Thymosin b4	Phase II
	Amniotic membrane extract eye drops	Phase I/II
	Mesenchymal stem cells	Phase I/II
Mucin secretagogues	Tavilermide (MIM-D3, 1% or 5%)	Phase II
	Ecabet sodium	Phase III
	Mycophenolate mofetil	Phase II
	15(s)-HETE or Icomucret	Phase III/II
Other’s products	Visomitin (SkQ1)	Phase II/III
	Tivanisiran (SYL1001)	Phase III

**Table 4:** CsA delivery from contact lenses for the management of DED.

Dosage Form	Contact Lens Material	Loading Method	Drug Release Duration	References
Contact lens	hydroxyethyl methacrylate (HEMA), cholesterol-hyaluronate (C-HA) micelle	mixing	12 days	[103]
Contact lens	poly-hydroxyethyl methacrylate (p-HEMA), Brij 97, Brij 78 and Brij 700	mixing	Brij 97—20 days, Brij 78—50 days, Brij 700—20 days	[86]
Silicone contact lenses	ethylene glycol dimethacrylate (EGDMA)	soaking	2 weeks, with vitamin E—1 month	[73]
Contact lens	poly-hydroxy ethyl methacrylate (p-HEMA), Brij 98	mixing	25 days	[87]
Contact lens	poly (2-hydroxyethyl methacrylate) (p-HEMA), Brij 97	mixing	20 days	[73]
Contact lens	graphene oxide	soaking	-	[104]

**Table 5:** The concentrations of major analytes in tears and their relative concentrations in the blood. Reprinted with permission from reference [114]. Copyright John Wiley and Sons.

Analyte	Tear Fluid Concentration [mM]	Blood Concentration [mM]	Diagnostic Application
Glucose	0.013–0.051	3.3–6.5	Diabetes management
Lactate	2.0–5.0	0.36–0.75	Ischemia, sepsis, liver disease, and cancer
Na^+^	120–165	130–145	Hyper/hyponatremia
K^+^	20–40	3.5–5.0	Hyper/hypokalemia and an indicator of ocular disease
Ca^2+^	0.4–1.1	2.0–2.6	Hyper/hypocalcemia
Mg^2+^	0.5–0.9	0.7–1.1	Hyper/hypomagnesemia
Cl^−^	118–135	95–125	Hyper/hypochloremia
HCO_3_^−^	20–26	24–30	Respiratory quotient indicator
Urea	3.0–6.0	3.3–6.5	Renal function
Pyruvate	0.05–0.35	0.1–0.2	Genetic disorders of mitochondrial energy metabolism
Ascorbate	0.22–1.31	0.04–0.06	Diabetes
Total Protein	≈7 g/L	≈70 g/L	Dry eye conditions, ocular insult, and inflammation
Dopamine	0.37	475 × 10^−9^	Glaucoma

**Table 6:** DED biomarkers identified in tear fluid.

Types of Biomarker Molecule	Biomarkers	References
Proteins	Lysozyme, lactoferrin, lysozyme proline-rich protein 4 (LPRR4), calgranulin A/S100 A8, lysozyme proline-rich protein 3 (LPRR3), nasopharyngeal carcinoma-associated PRP 4, α-1 antitrypsin α-enolase, α-1 acid glycoprotein 1, S100 A4, S100 A11 (calgizzarin), S100 A9/calgranulin B, lipocalin-1 (LCN-1), mammaglobin B, lipophilin A, beta-2 microglobulin (B2M), S100A6, annexin A1 annexin A11, cystatin S (CST4), phospholipase A2-activating protein (PLAA), transferrin, defensin-1, clusterin, lactotransferrin, cathepsin S, anti-SS-A, anti-SS-B, anti-α-fodrin antibodies, malate dehydrogenase (MDH) 2, palate lung nasal clone-PLUNC	[115,121]
Mucins	(MUC)5AC	[122]
Neuromediators	Nerve growth factor (NGF), calcitonin gene related peptide (CGRP), neuropeptide Y (NPY), vasointestinal peptide (VIP), serotonin, substance P	[119]
Cytokines/chemokines	Interleukin-1(IL-1), interleukin-2 (IL-2), interleukin-5 (IL-5), interleukin-6 (IL-6), interleukin 8 (IL-8) or chemokine (C-X-C motif) ligand 8 (CXCL8), interleukin-10 (IL-10), interleukin-12 (IL-12), interleukin-16 (IL-16), interleukin-33 (IL-33), GCSF, monocyte chemoattractant protein 1 (MCP1)/chemokine (C-C motif) ligand 2 (CCL2), MIP5/chemokine (C-C motif) ligand 15 (CCL15), C-X-C motif chemokine 5 (CXCL5 or ENA78), soluble interleukin-1 receptor Type I (sIL-1RI), soluble interleukin-6 receptor (sIL-6R), soluble gp130 (sgp130), soluble vascular endothelial growth factor receptor 1 (sVEGFR1), soluble epidermal growth factor receptor (sEGFR), soluble tumor necrosis factor receptor I (sTNFR I), interleukin-17A (IL-17A), interleukin-21 (IL-21), interleukin-22 (IL-22), interleukin-1 receptor antagonist (IL-1RA), chemokine (C-X-C motif) ligand 9 (CXCL9)/monokine induced by gamma interferon (MIG), interferon-inducible T-cell alpha chemoattractant (I-TAC)/C-X-C motif chemokine 11 (CXCL11), C–X–C motif chemokine 10 (CXCL10)/interferon γ-induced protein 10 kDa (IP-10), ligand 4 (CCL4)/macrophage inflammatory protein-1β (MIP-1β), chemokine (C-C motif) ligand 5 (also CCL5)/regulated on activation, normal T cell expressed and secreted (RANTES), epidermal growth factor (EGF), tumor necrosis factor alpha (TNF-α), interferon gamma (IFNγ), matrix metallopeptidase 9 (MMP-9), macrophage inflammatory protein-1 alpha (MIP-1α/CCL3), vascular endothelial growth factor (VEGF), fractalkine	[115,123,124]
Lipids	(O-acyl) ω-hydroxy fatty acids (OAHFAs), lysophospholipids, PUFA-containing diacylglyceride species, hexanoyl-lysine (HEL), 4-hydroxy-2-nonenal (HNE), malondialdehyde (MDA)	[125]
Metabolites	Cholesterol, N-acetylglucosamine, glutamate, creatine, amino-n-butyrate, choline, acetylcholine, arginine, phosphoethanolamine, glucose, phenylalanine	[126]
Tear solutes	Osmolarity	[127]

**Table 7:** Electrochemical sensors that can sense vital biomarkers of DED in tears. Adapted from reference [24]. Copyright John Wiley and Sons.

Biomarkers	Sensor Type
Glucose	Enzymatic biosensor; amperometric
Osmolarity	Impedimetric
MMP-9	Electrochemical immunosensors
Urea	Voltammetric
Serum	Electrochemical immunosensors
TNF-α	Electrochemical aptamer sensor
Mucins	Electrochemical immunosensors
